# COVID-19 Vaccination Intentions: The Theory of Planned Behavior, Optimistic Bias, and Anticipated Regret

**DOI:** 10.3389/fpsyg.2021.648289

**Published:** 2021-06-16

**Authors:** Katharina Wolff

**Affiliations:** Department of Psychosocial Science, Faculty of Psychology, University of Bergen, Bergen, Norway

**Keywords:** COVID-19 vaccination, Theory of planned behavior, optimistic bias, anticipated regret, vaccination uptake

## Abstract

High vaccination rates within the general population are essential for overcoming the current COVID-19 pandemic. The aim of the present study was to investigate intentions to receive a COVID-19 vaccine as well as the predictors of such intentions. A representative sample of the Norwegian population (*N* = 1,003, 49.5% females, M_age_ = 47.9, *SD* = 17.1) filled in an online questionnaire assessing the components of the Theory of planned behavior (attitudes, subjective norms, and perceived behavioral control), as well as optimistic bias and anticipated regret. Results showed that a majority (61.6%) of participants intend to get vaccinated. Regression analysis revealed that intentions were predicted by positive attitudes toward vaccination (β = 0.31, *p* < 0.001), subjective norms in favor of vaccination in one’s family (β = 0.23, *p* < 0.001), perceived behavioral control (β = 0.09, *p* < 0.001), and by anticipated net regret (β = 0.32, *p* < 0.001), explaining 69% (*f*^2^ = 2.23) of the variance in intentions. Optimistic bias did not predict intentions.

## Introduction

The Coronavirus outbreak was declared a pandemic by the WHO in March 2020. By December 2020, the disease had caused over 1.8 million death (WHO, 2020b) and the largest global recession since the great depression (Financial Times, 2020). At the moment numerous COVID-19 vaccine candidates are being tested and several have been or are at the verge of being approved for use in the general population (WHO, 2020a). Vaccines have the potential of saving millions of lives, and vaccination uptake is crucial to succeed in combating the Coronavirus disease.

Previous research has identified various factors influencing vaccination intentions and vaccination uptake including socioeconomic factors like higher income and higher education (e.g., Jain et al., 2017), being Caucasian and holding health insurance (e.g., Fisher et al., 2013 for HPV-vaccination) as well as psychological factors like perceived risk, susceptibility, and severity (e.g., Brewer et al., 2007 for adult vaccination against infectious disease).

Some factors influencing intentions to vaccinate against COVID-19 have also been identified. These findings are, however, less consistent. Kwok et al. (2021) found that younger age, more confidence and collective responsibility, and less complacency in Hong Kong nurses predicted willingness to be vaccinated. Malik et al. (2020) found that older age, being male, Asian, and more educated correlated with vaccination acceptance in a US sample. Sherman et al. (2020) found the following predictors of vaccination intentions in a sample of United Kingdom adults: more positive COVID-19 vaccination beliefs and attitudes, less concerns regarding vaccination side effects, greater perceived information sufficiency to make an informed decision about COVID-19 vaccination, increased risk perceptions of COVID-19 to others (but not risk to oneself), older age, and having been vaccinated for influenza last winter (2019/20). Vaccination rejection correlated with the belief the threat of COVID-19 has been exaggerated, and with inadequate health literacy and lower education in an Australian study (Dodd et al., 2021) and with mistrust of vaccine benefit, worry about unforeseen future effects, concerns about commercial profiteering from pharmaceutical companies, and preferences for natural immunity in a North American study (Taylor et al., 2020).

The present investigation is theoretically driven and set out to assess intentions to get vaccinated against the Coronavirus as well as psychological predictors of such intentions in a representative sample of the Norwegian population before any vaccine became available. We examined whether the Theory of planned behavior (TPB, Ajzen, 1985, 1991), optimistic bias, and anticipated regret regarding vaccination could predict intentions to get vaccinated.

The TPB is an expectancy-value model used to predict and explain human behavior in specific contexts (Ajzen, 1985, 1991). Its utility has been demonstrated in predicting various health related behaviors including intentions to obtain genetic testing (Wolff et al., 2011), intentions to vaccinate against COVID-19 (Sherman et al., 2020) and actual vaccine uptake (Gerend and Shepherd, 2012). According to the TPB intentions are the direct precursor of behavior, and are in turn determined by attitudes, subjective norms, and perceived behavioral control. Attitudes are defined as the individual’s cognitive and affective evaluation of a given behavior as either positive or negative. Subjective norms represent the individual’s perceived social pressure to perform that behavior and consist of injunctive norms (describing how people should act) and descriptive norms (describing how people actually act). Perceived behavioral control refers to the individual’s beliefs about being able to perform the behavior.

Optimistic bias describes people’s tendency to overestimate the probability of experiencing positive events and underestimating the probability of experiencing negative events, compared to others (Weinstein, 1980, 1983, 1989). Hence people overestimate their chances for good health and underestimate their chances of getting ill. It has been suggested that optimistic bias may influence intentions to get vaccinated negatively in that optimistically biased people are less willing to get vaccinated (Bond and Nolan, 2011; Dubov and Phung, 2015). It has also been shown that increased perceptions of vulnerability and risk regarding the disease correlate with positive attitudes toward vaccination (Timmermans et al., 2008) and vaccination uptake (Weinstein et al., 2007).

Anticipated regret is another potential predictor of intentions to vaccinate against COVID-19. According to Regret theory (Bell, 1982, 1985; Loomes and Sugden, 1982, 1987) people anticipate the feelings they might experience once the outcome of a decision becomes apparent. Foreseeing possible unwanted outcomes of an alternative may lead decision makers to anticipate regret and shun that option. A meta-analysis (Brewer et al., 2016) has shown anticipated regret to affect various types of health behaviors including vaccination. For vaccination, the analysis showed that ratings of anticipated regret from vaccination were generally lower than ratings of anticipated regret from not vaccinating. This might be explained by the fact that people anticipate less regret and self-blame for easily justifiable decisions than for less justifiable ones (Zeelenberg and Pieters, 2007).

The present study investigates the following hypothesis: Intentions to vaccinate against the Coronavirus correlate positively with positive attitude toward vaccination, with perceived subjective norms in favor of vaccination among friends and family, and with high perceived behavioral control. Furthermore, intentions are predicted by increased perceived relative susceptibility and seriousness (i.e., lower optimistic bias), as well as by lower anticipated regret for vaccination, and higher anticipated regret for not vaccinating.

## Materials and Methods

### Participants and Procedure

A link to an online questionnaire was sent to a representative sample of the Norwegian population above the age of majority (age 18 and older) either by e-mail or via a smartphone-app. Respondents were a random sample stratified according to age, gender, and geographical region drawn from a panel of 80,000 Norwegians. Data collection was done by NORSTAT (a large commercial European data collector). Data were not weighted for representativeness. Data collection took place during the first 3 weeks of December 2020 and lasted until a number of 1,000 completed questionnaires was reached (*N* = 1003). Males constituted 49.5% of the participants (*N* = 496), mean age was 47.9 (*SD* = 17.1) (range: 18–87). The response rate was 32%, dropout rate 7%. It took about 5–6 min to fill in the questionnaire.

### Ethics Statement

Ethical review and approval were not required for the study on human participants in accordance with the local legislation and institutional requirements. The author of the paper still deemed the project to be within the requirements of the Helsinki declaration (World Medical Association, 2013).

Participants were informed that the study was on Corona vaccination; that participation implied consent, was voluntary and could be stopped at any time; and that data were collected anonymously.

### Measures

All questionnaire items were constructed for the purpose of the present investigation and in line with previous research. Items were presented in the same order they are described and presented in the following. TPB-variables were assessed by items constructed in accordance with Ajzen (2006) instructions. All items were measured on 7-point bipolar scales. Behavioral intentions were measured by two items: *If a vaccine against Corona becomes available, I will vaccinate myself./I will take a vaccine against Corona when it will be offered.* anchored at *very improbable* (1) and *very probable* (7). Scores were averaged to constitute a measure of intention (*r* = 0.94; *p* < 0.001).

Attitudes were measured by seven semantic differentials, including both cognitive and affective evaluations of vaccination. *To take a vaccine against Corona is: bad-good; stupid-wise; dangerous-safe; useless-effective; unpleasant-pleasant; irresponsible-responsible; disturbing-reassuring.* Scores were averaged to constitute a measure of attitude (α = 0.92).

Injunctive and descriptive subjective norms for friends and family were assessed by four items. *What do your friends (your closest family) think of you taking a Corona vaccine?* anchored by *very much against it* (1) and *very much for it* (7). *Most of my friends (my closest family) will take a Corona vaccine themselves*, anchored by *not correct* (1) and *correct* (7). Items for *friends* (*r* = 0.82; *p* < 0.001) and for *family* (*r* = 0.87; *p* < 0.001) were averaged.

Perceived behavioral control was measured by two items assessing capacity and autonomy. *If a vaccine becomes available, I will be able to get vaccinated./If a vaccine becomes available, It is up to me whether I get vaccinated or not*, anchored by *not correct* (1) and *correct* (7). Since items correlated only moderately (*r* = 0.23; *p* < 0.001) they were not averaged as planned but entered separately into the analysis. This was done despite the disadvantages of using one-item-measures.

Optimistic bias was measured by two items assessing perceived relative susceptibility and perceived relative probability of a serious prognosis for the participant compared to a reference group. This is in accordance with Weinstein (1980) way of assessing unrealistic optimism. *Compared to other Norwegians your own age, what is the likelihood that you will be infected with Corona?* (relative susceptibility)/*Compared to other Norwegians your own age, what is the likelihood that you would experience a serious course of a Corona infection*, (relative seriousness of prognosis) anchored by *much lower* (1) and *much higher* (7). Items should not be expected to correlate as susceptibility and prognosis of COVID-19 are not known to correlate, however, there was a moderate positive correlation (*r* = 0.32; *p* < 0.001). Items were reversed and averaged to constitute a measure of optimistic bias.

Anticipated regret was measured by two items. *If I take a Corona vaccine, I might regret it./If I do NOT take a Corona vaccine, I might regret it.* anchored by *very improbable* (1) and *very probable* (7). The score on the first item was subtracted from the score of the second item to achieve a measure of net-anticipated regret (*r* = -0.54; *p* < 0.001). Item construction was in accordance with Brewer et al. (2016) specification of anticipated regret measures.

### Analysis Plan

All analyses were run using IBM SPSS (Version 25). A two-step hierarchical regression analysis was run to test for predictors of vaccination intentions. In the first step (Model 1) demographic variables and Theory-of-planned-behavior variables were entered. In the second step (Model 2) perceived relative susceptibility and seriousness, as well as anticipated regret for getting vaccinated and for NOT getting vaccinated were entered. This order was chosen to investigate whether entering additional variables could improve the predictive power of the TPB. A separate regression analysis was run keeping only significant predictors, and replacing anticipated regret for getting vaccinated and for NOT getting vaccinated by the compound measure of net-regret (Model 3).

## Results

A clear majority of respondents (61.6%) indicate that they intend to get vaccinated against the Coronavirus (scores above 5 on a scale from 1 to 7). 13.8% of participants indicate that they do not intend to get vaccinated (scores below 3) and 24.8% are uncertain (scores between 3 and 5). Table 1 presents means and standard deviations for vaccination intention for various age groups and for men and women. Table 2 displays means, standard deviations, and correlations for all variables.

**TABLE 1:** Means and standard deviations for vaccination intention for various age groups and for men and women.

Age	18–29 (*n* = 185)		30–39 (*n* = 175)		40–49 (*n* = 182)		50–59 (*n* = 195)		60–69 (*n* = 122)		70–79 (*n* = 130)		> 80 (*n* = 14)	
	M	SD	M	SD	M	SD	M	SD	M	SD	M	SD	M	SD
	5.25	0.15	4.79	0.15	5.15	0.16	5.23	0.14	5.96	0.15	6.16	0.13	6.61	0.36

**Gender**	**Men (*n* = 496)**		**Women (*n* = 507)**											
	**M**	**SD**	**M**	**SD**										
		
	5.61	0.08	5.14	0.09										

**TABLE 2:** Means, standard deviation, and Pearson correlation matrix for all variables.

	M	SD	1	2	3	4	5	6	7	8	9	10	11	12	13
1. Intention	5.37	1.95	–												
2. Age	47.92	17.10	0.19*	–											
3. Female gender	–	–	−0.12*	−17*	–										
4. Attitude	5.51	1.27	0.75*	0.14*	−0.10*	–									
5. Social norm Friends	5.34	1.40	0.68*	0.10*	−0.08*	0.68*	–								
6. Social norm Family	5.64	1.47	0.72*	0.14*	−0.11*	0.70*	0.80*	–							
7. Perceived control (capability)	5.59	1.63	0.51*	0.23*	–0.08	0.48*	0.47*	0.48*	–						
8. Perceived control (autonomy)	6.10	1.43	0.09*	0.15*	–0.07	0.13*	0.10*	0.10*	0.23*	–					
9. Relative susceptibility	3.73	1.22	0.16*	0.04	0.04	0.12*	0.12*	0.13*	0.14*	–0.03	–				
10. Relative seriousness	3.86	1.47	0.24*	0.34*	0.02	0.20*	0.14*	0.17*	0.27*	0.02	0.32*	–			
11. Optimism bias	−3.79	1.10	−0.25*	−0.25*	–0.03	−0.20*	−0.16*	−0.18*	−0.26*	0.01	−0.77*	−0.85*	–		
12. Anticipated regret vaccination	2.96	1.69	−0.61*	−0.15*	0.12*	−0.60*	−0.52*	−0.54*	−0.36*	0.10*	0.01	–0.08	0.05	–	
13. Anticipated regret no vaccination	5.22	1.66	0.70*	0.16*	–0.04	0.62*	0.61*	0.64*	0.45*	0.02	0.16*	0.30*	−0.29*	−0.54*	–
14. Net anticipated regret	2.26	2.94	0.74*	0.18*	−0.09*	0.70*	0.64*	0.67*	0.46*	0.07	0.09*	0.21*	−0.19*	−0.88*	0.88*

*p < 0.001.

Results of the regression analysis are presented in Table 3. In the first step of the analysis (Model 1) demographic and TPB variables were entered. These variables explained 66% of the variance in intentions. Results showed that intentions to vaccinate correlate with the variables of the TPB, the strongest predictor being positive attitudes, followed by perceived social norms within one’s family, and among one’s friends. Of the perceived behavioral control measures only perceived capability, but not perceived autonomy predicted intentions. In addition, there was a very weak correlation with age (which is also a risk factor for a serious prognosis of COVID-19). In the second step of the analysis (Model 2) perceived relative susceptibility and seriousness, and anticipated regret were added, increasing the explained variance to 70%. Results showed that both anticipated regret regarding getting vaccinated and anticipated regret regarding NOT getting vaccinated predict behavioral intentions. Both are quite strong predictors, rendering several other variables insignificant [i.e., social norms among friends; perceived behavioral control (autonomy) and age]. Neither relative susceptibility nor seriousness predicted intentions to vaccinate (due to collinearity optimistic bias was not entered into the regression model together with perceived susceptibility and seriousness. Entering optimistic bias instead of these variables yields parallel results. Despite a negative bivariate correlation between optimistic bias and intentions to vaccinate, optimistic bias does not predict intentions in the regression model).

**TABLE 3:** Two-step hierarchical regression analysis (Model 1 and 2) and separate regression containing significant predictors (Model 3) of intention to get vaccinated.

	B	95% CI	SEB	β	R^2^	ΔR^2^
**Model 1**					0.66**	0.66**
Age	0.01	[0.0, 0.01]	0.00	0.05*		
Female gender	–0.10	[−0.24, 0.05]	0.07	–0.03		
Attitude	0.66	[0.57, 0.74]	0.04	0.43**		
Social norm Friends	0.18	[0.09, 0.27]	0.05	0.13**		
Social norm Family	0.33	[0.24, 0.42]	0.05	0.25**		
Perceived control (capability)	0.14	[0.09, 0.19]	0.03	0.12**		
Perceived control (autonomy)	–0.06	[−0.11, -0.01]	0.03	–0.04		
**Model 2**					0.70**	0.05**
Age	0.00	[−0.0, 0.01]	0.00	0.02		
Female gender	–0.13	[−0.27, 0.01]	0.07	–0.03		
Attitude	0.47	[0.38, 0.55]	0.04	0.30**		
Social norm Friends	0.11	[0.03, 0.20]	0.04	0.08		
Social norm Family	0.23	[0.15, 0.32]	0.04	0.17**		
Perceived control (capability)	0.09	[0.04, 0.14]	0.03	0.08**		
Perceived control (autonomy)	–0.03	[−0.08, 0.02]	0.03	–0.02		
Relative susceptibility	0.06	[−0.00, 0.12]	0.03	0.04		
Relative seriousness	0.03	[−0.02, 0.09]	0.03	0.03		
Anticipated regret vaccination	–0.16	[−0.22, -0.11]	0.03	−0.14**		
Anticipated regret no vaccination	0.26	[0.20, 0.32]	0.03	0.22**		
**Model 3**					0.69**	
Attitude	0.48	[0.40, 0.57]	0.04	0.31**		
Social norm Family	0.31	[0.24, 0.38]	0.04	0.23**		
Perceived control (capability)	0.11	[0.06, 0.16]	0.03	0.09**		
Net anticipated regret (no vaccination – vaccination)	0.22	[0.18, 0.25]	0.02	0.32**		

*p < 0.01, **p < 0.001.

Retaining only the significant predictors in the regression analysis (Model 3) and using net-anticipated regret (anticipated regret no vaccination – anticipated regret vaccination) instead of both regret measures showed that 69% of the variance in intentions to get vaccinated could be explained by four variables: attitudes, social norms within one’s family, perceived capability (all TPB variables), and net-anticipated regret. Net-anticipated regret and attitudes were the strongest predictors of intentions, followed by norms in one’s family and perceived capability [keeping both measures of anticipated regret (for getting vaccinated and for NOT getting vaccinated) in the analysis yields parallel results. For parsimony we therefore included net-anticipated regret].

## Discussion

Results reveal that a majority (61.6%) of Norwegians is willing to be vaccinated against the Coronavirus, 24.8% of the population is uncertain, and 13.8% indicate that they do not intend to receive a vaccine. Findings are comparable to Brewer et al. (2007) who in a meta-analysis of vaccination behavior for various infectious diseases found vaccination rates to vary between 6 and 86% (with a median uptake of 51%). Vaccination rates for seasonal influenza in Norway are somewhat lower than the reported intentions in the present study, about 38% for those above the age of 65 in 2019 (OECD, 2021). Intentions to get vaccinated are mainly predicted by positive attitudes toward vaccination as well as the degree to which anticipated regret for non-vaccination outweighs anticipated regret for vaccination. Another predictor of intentions was one measure of perceived behavioral control, i.e., perceived capability, but not perceived autonomy. Subjective norms toward vaccination within one’s family, but not among one’s friends predicted vaccination intentions. Together these variables explain 69% of the variance in intentions.

Neither perceived relative susceptibility nor seriousness, nor optimistic bias predicted intentions to vaccinate. This is in line with Sherman et al.’s (2020) findings which showed that participants were more willing to vaccinate against COVID-19 when they perceived greater risk for others, but not for themselves. Findings are, however, in contrast with other research that has found increased risk perceptions and increased vulnerability to predict protective health behaviors including vaccination (Brewer et al., 2007). Since COVID-19 may not constitute a significant risk for the majority of respondents, vaccination intentions might be predicted by a motivation to protect others rather than a motivation to protect oneself. This would contrast with other diseases where research has found that increased perceived vulnerability does predict vaccination uptake (Brewer et al., 2007). Note also that we measured relative susceptibility and seriousness, i.e., perceived vulnerability compared to that of others in one’s age group. We therefore do not know whether absolute perceived susceptibility and seriousness are high or low in the population. These variables were assessed this way to obtain a measure of optimistic bias, as this has been suggested to predict vaccination (Bond and Nolan, 2011; Dubov and Phung, 2015). Optimistic bias, however, did not predict vaccination intentions in the regression model. This finding would be expected if vaccination against COVID-19 is motivated by the protection of others, not oneself. Optimistic bias may still play a role in vaccination behavior regarding other diseases.

Anticipated regret was found to be one of the strongest predictors of intentions to vaccinate. This is in line with previous research, and as in previous research, it was found that anticipated regret was lower for getting vaccinated (action regret) than for NOT getting vaccinated (inaction regret) (Brewer et al., 2016). As pointed out earlier, this might be explained by the fact that people anticipate less regret and self-blame for easily justifiable decisions (e.g., virtues, health promoting behavior) than for less justifiable ones (e.g., vices, risk behavior) (Zeelenberg and Pieters, 2007). It was also found that net-regret (the degree to which anticipated regret for NOT getting vaccinated outweighed regret for getting vaccinated) was the strongest predictor of intentions, followed by positive attitudes toward vaccination.

It is also interesting to note that anticipated regret for getting vaccinated and for NOT getting vaccinated show a moderate negative correlation. To the degree that anticipated regret is determined by the possibility of negative outcomes of the chosen option, regret for getting vaccinated and for NOT getting vaccinated should correlate positively. This is because the greater the disadvantages of not vaccinating the population are, the greater side effects of vaccination will be accepted by society. The paradoxical negative correlation in the population could be explained by the affect heuristic (Finucane et al., 2000; Slovic et al., 2007). According to this heuristic people use their affective reaction toward a stimulus to judge its’ risk and benefits. Liking something leads to an evaluation of that stimulus as low in risk and high in benefits, while disliking something leads to an evaluation of the stimulus as high in risk and low in benefits. In this way risks and benefits end up being negatively correlated in people’s minds, even though they are positively related in the real world. That is, society accepts high risks technologies or activities only if benefits are high as well (Finucane et al., 2000).

If the affect heuristic influences participants’ judgments of the given vaccination alternatives (getting vaccinated or NOT getting vaccinated), this might lead them to downplay the risks and exaggerate the benefits of the preferred alternative, and as the data seem to indicate, to exaggerate the risk and downplay the benefits of the non-chosen option. In other words, increasing people’s anticipated regret for NOT getting vaccinated might decrease their anticipated regret for getting vaccinated. This is of course purely speculative and would need to be tested in future research.

There are of course several limitations of the present investigation. The main weakness being that only intentions were measured instead of actual behavior. In the present case it was not possible to measure behavior, since data were collected before any vaccines became available in Norway. Another limitation is the fact that one-item-measures were used to assess perceived capability and perceived autonomy. These items were planned as a compound measure of perceived behavioral control but did not correlate. Furthermore, perceived vulnerability was measured in relative (compared to others) not absolute terms. Therefore, we do not know whether the perceived risk of COVID-19 is high or low in the population. Measures were constructed this way to assess optimistic bias. In hindsight, COVID-19 may not be a disease that lends itself well to assessing whether optimistically biased people are less inclined to get vaccinated, simply because the disease does not constitute a significant risk for most people, at least not in Norway (Folkehelseinstituttet, 2020).

Summing up, results indicate that intentions to vaccinate against COVID-19 are predicted by low anticipated regret following vaccination and high anticipated regret following non-vaccination, by positive attitudes toward vaccination, by perceived social norms in favor of vaccination within one’s family, and to a small extend by perceived capability. Interventions to increase vaccination uptake should focus on these variables. Increasing positive attitudes toward vaccination may be achieved by information about vaccine benefits, however, increasing anticipated regret for non-vaccination by focusing on the disadvantages of not getting vaccinated may be as effective. Results even indicate that increasing non-vaccination regret might decrease vaccination regret. More research is needed to test this assumption. Results also showed that intentions to get vaccinated were not predicted by increased perceived susceptibility or seriousness, nor by optimistic bias. This may be because vaccination against this particular disease is predicted by a motivation to protect others, more than oneself. If this is the case, interventions aimed at increasing vaccination uptake for COVID-19 should not focus too much on how the disease may harm the individual, but rather on how the disease may harm others, like elderly family members, or society at large, and on how high vaccination rates will protect those that are at risk. Focusing on benefits of vaccination for older family members may also increase social norms in favor of vaccination within one’s family, which is another potential predictor of vaccination uptake. Furthermore, the results also indicate that the TPB explains a large proportion of intentions to get vaccinated. Still, the TPBs’ predictive power was further improved by including measures of anticipated regret. This is in line with other research, including a meta-analysis by Sandberg and Conner (2008).

Future research should aim at measuring actual vaccination behavior instead of intentions only. More research is needed to establish the effects of increased risk perceptions for others (not oneself) on vaccination intentions and behavior. It would also be interesting to investigate whether there is a negative causal relation between anticipated regret for the chosen and the non-chosen option as this would be an illogical relation.

## Data Availability Statement

The raw data supporting the conclusions of this article will be made available by the author, without undue reservation, to any qualified researcher.

## Ethics Statement

Ethical review and approval was not required for the study on human participants in accordance with the local legislation and institutional requirements. Written informed consent for participation was not required for this study in accordance with the national legislation and the institutional requirements.

## Author Contributions

The author confirms being the sole contributor of this work and has approved it for publication.

## Conflict of Interest

The author declares that the research was conducted in the absence of any commercial or financial relationships that could be construed as a potential conflict of interest.
